# Regaining control over alcohol intake but not abstinence on disulfiram medication, as a harm reduction approach: 2 case reports

**DOI:** 10.1186/s13722-024-00522-1

**Published:** 2024-12-02

**Authors:** Max Schallenberg, Maximilian Pilhatsch, Johannes Petzold, Diana Vogel-Blaschka, Ulrich S. Zimmermann, Maik Spreer

**Affiliations:** 1 Department of Psychiatry and Psychotherapy, Carl Gustav Carus Faculty of Medicine, Carl Gustav Carus University Hospital Dresden, Dresden University of Technology, Dresden, Germany; 2 Department of Psychiatry and Psychotherapy, Elblandklinikum Radebeul, Radebeul, Germany; 3 Department of Addiction Medicine and Psychotherapy, kbo Isar-Amper-Klinikum Region München, Haar, Germany

**Keywords:** Disulfiram, Alcohol use disorder, Alcohol dependence treatment, Harm reduction, Reduced drinking

## Abstract

**Background:**

Alcohol use disorder (AUD) poses severe health risks, yet many affected individuals opt out of complete abstinence. Therefore, harm reduction strategies have become more prominent in treatment guidelines for AUD. Our two case reports illustrate how disulfiram, initially intended to enforce abstinence, was repurposed to support reduced drinking.

**Case Presentations:**

A 41-year-old patient with a history of severe AUD successfully reduced his alcohol consumption to a low-risk level by leveraging the effects of the disulfiram-alcohol aversive reaction. Another patient, a 63-year-old woman with long histories of AUD and major depressive disorder, experienced fewer depressive episodes and hospitalizations with disulfiram therapy despite periodically intentional discontinuation of medication.

**Conclusion:**

Individualized treatment strategies are critical in optimizing outcomes for patients with AUD. Continuous disulfiram therapy, despite its limitations in directly reducing alcohol intake, might offer a new avenue for harm reduction in exceptional cases even if alcohol consumption continues. The cases suggest that maintaining therapy, aiming at reduced drinking, can enhance the therapeutic alliance and help manage comorbid conditions. Regular medical monitoring is essential for safety and efficacy, warranting further study of possible long-term consequences and psychotropic effects of elevated acetaldehyde levels related to the disulfiram-alcohol interaction.

## Background

Alcohol use presents a major health hazard, contributing substantially to mortality and morbidity rates worldwide. Although a range of treatments for alcohol use disorder (AUD) exists, treatment rates are generally low [1]. Many individuals with AUD are hesitant to engage in treatment, nearly half of them due to unwillingness to cease alcohol consumption entirely [1]. Estimates suggest that 20–80% of patients with an alcohol dependence prefer reduced-risk drinking (RRD) over total abstinence, highlighting the importance of addressing this preference to narrow the treatment gap [2, 3].

The harm reduction approach has gained acceptance by various clinical guidelines, such as those from the National Institute of Health and Care Excellence (NICE), the American Psychiatric Association (APA), and the French [4] and German guidelines [5], particularly when the abstinence approach is not feasible. The European Medicine Agency (EMA) suggests that a reduction of at least 2 categories in the World Health Organization (WHO) drinking risk levels significantly benefits individuals’ health [6]. Benefits of even modest reductions in alcohol consumption include prompt improvement in nutritional deficiencies and blood pressure [7], along with reduced risks of mortality [8], cirrhosis [9], cancer, cardiovascular diseases [7], osteoporosis [10], and pancreatitis [11]. Such outcomes also lessen the economic burden associated with alcoholism [12–15].

Historically, medications approved for AUD, such as disulfiram – the first and for decades the sole medication approved for alcohol dependence treatment – focused on promoting abstinence. Since the 1990s, research has increasingly focused on reducing drinking levels as a measure of medication efficacy, aligning with the principles of “harm reduction”.

Approved medications such as oral naltrexone and nalmefene [16] are used for this purpose in clinical settings. Gabapentin, topiramate, and baclofen have also reduced alcohol intake in numerous studies and are therefore often used off-label in clinical practice [17–19].

Despite these advances, disulfiram remains unique in its mechanism and therapeutic objective of total abstinence. Its inhibition of the acetaldehyde dehydrogenase induces an accumulation of acetaldehyde upon alcohol consumption, which leads to an potentially severe and life-threatening aversive reaction intended to serve as a deterrent to prevent any alcohol intake [20]. The ensuing case presentations delineate two patients for whom disulfiram therapy diverges from its conventional goal of promoting abstinence to instead pursue a “Harm Reduction” approach.

### Case presentations

Both patients provided written informed consent after receiving comprehensive information about therapy. The treatment was administered in an outpatient setting, which is situated within the psychiatric hospital.

#### Patient 1

A 41-year-old Caucasian man with a history of alcohol exposure from age 16 developed AUD by age 31. The patient experienced complications, such as withdrawal-induced seizures, as well as co-occurring mental health conditions like post-traumatic stress disorder (PTSD), recurrent depression, and other substance use disorders. He did not achieve stable abstinence under acamprosate or naltrexone, reflected by his treatment history of five inpatient detoxifications for alcohol, numerous premature terminations and non-attendance at appointments. The average daily alcohol consumption was 236.8 g of pure ethanol, with the highest documented blood alcohol concentration reaching 3.6 g/l. The patient was prescribed 300 mg of venlafaxine, 300 mg of trazodone, and 200 mg of quetiapine per day.

Due to the lack of therapeutic success, disulfiram treatment was initiated for the first time in February 2022. The disulfiram programme was initiated with a low dose, with the objective of assessing tolerance. This dose was then increased gradually until the desired effect was achieved. Both patients exhibited no adverse drug reactions (ADR) when consuming alcohol while on low doses of disulfiram. Disulfiram 750 mg was administered three times weekly under personal supervision, at the outpatient clinic, which is situated within the psychiatric hospital. This common treatment regimen in Germany is a compromise between safety aspects, clinical practicability and personal autonomy of patients [21]. The treatment regimen was primarily based on the recommendations set forth by Zimmermann et al. (2021) [22]. These are similarly employed in Germany as part of the Network of Alcohol-Aversive Pharmacotherapy (NAP) [21]. The dosage of 750 mg three times a week is also consistent with the range of 125–500 mg/d recommended by the FDA [23–25]. The supervised intake process guarantees the regular and controlled administration of medication, while the long half-life of disulfiram guarantees the effective concentration of the active ingredient. Additionally, patients are only required to make the decision to abstain or continue treatment three times a week, which could result in greater adherence to the treatment plan and longer retention in the treatment process [21]. Throughout the treatment period, the patient repeatedly tested positive for ethyl glucuronide in his urine, indicating alcohol consumption. Upon confrontation, he reported that he drank small quantities of alcohol despite regular clinic visits while adhering to disulfiram intake. The resulting aversive reactions were mild, primarily involving facial flushing and slight nausea, and were insufficient to deter him from drinking but prevented him from instances of loss of control. After the patient opted against increasing the dose of disulfiram, a careful consideration of the risks and benefits led to the continuation rather than termination of disulfiram treatment.

Despite ongoing alcohol use, disulfiram helped regulate his consumption, preventing loss of control. His alcohol intake decreased to 40 g/day, marking a shift from high-risk to low-risk consumption [17]. This pharmacological approach facilitated engagement in psychotherapeutic care, and the patient maintained abstinence from opiates and benzodiazepines. The patient has maintained stability under this regimen for the past two years and continues receiving treatment.

#### Patient 2

A 63-year-old unmarried Caucasian woman had an extensive history of AUD with several sequelae and frequent sick leaves, threatening her professional career as a teacher. Her alcohol use began at age 15, escalating to AUD by age 43. Notably, her highest recorded blood alcohol concentration was 4.13 g/l.

She successfully quit smoking in 2017 and had no history of illegal drug use, but a family history of AUD was noted, with both her father and brother being affected.

The patient’s mental health was further complicated by a recurrent depressive disorder, which was exacerbated by alcohol consumption, frequently leading to suicidal ideation. One suicide attempt involving lithium intoxication resulted in admission to an intensive care unit.

Moreover, she suffered from multiple sclerosis and arterial hypertension.

Over the years, she underwent four inpatient detoxifications, three long-term inpatient rehabilitations, and a four-month outpatient rehabilitation. As these treatments including adequate attempts with naltrexone remained unsuccessful, disulfiram treatment was initiated in November 2014 and well tolerated except for minor gastrointestinal complaints at the beginning. Previously to disulfiram initiation her liver parameters were slightly elevated alanine aminotransferase (ALAT) 1,14 µmol/s*l, aspartate aminotransferase (ASAT) 0,87 µmol/s*l). Disulfiram 750 mg was administered three times weekly under direct supervision at the outpatient clinic, the outpatient clinic is situated within the psychiatric hospital. The disulfiram treatment was conducted in accordance with the identical treatment plan employed for patient 1 [21]. Daily medications were amlodipine (5 mg), candesartan (8 mg), duloxetine (60 mg), lithium carbonate (900 mg), melperone (25 mg) and ocrelizumab (600 mg). The patient participated in a self-care group and received individual psychotherapy.

The patient remained abstinent for the first eight months following disulfiram initiation but reported occasional deliberate relapses during the subsequent treatment course. These relapses were often triggered by social events yet were managed effectively through regaining adherence to disulfiram. She deliberately discontinued disulfiram for at least three days prior to these relapses, which were characterized by consuming approximately 1 L of wine and mild aversive reactions with palpitations and facial flushing.

Although the disulfiram therapy did not achieve sustained abstinence, alcohol consumption significantly decreased from an average of 208 g per day before disulfiram treatment to 100 g per quarter of a year, which represents a transition from high-risk to low-risk alcohol consumption [17]. Following the initiation of disulfiram treatment, liver parameters returned to their normal range (ALAT 0,38 µmol/s*l, ASAT 0,52 µmol/s*l) in year 2018. There was no further depressive decompensation, and hospitalization was no longer necessary. She is still undergoing treatment.

## Discussion and conclusion

Our two case reports describe the continuation of disulfiram therapy despite patients’ ongoing, either persistent or intermittent, alcohol consumption, without altering the dosage regimen. Although, cessation of disulfiram treatment is advised under such circumstances to prevent life-threatening complications [14, 26, 27], we continued disulfiram treatment after detailed risk-benefit assessments as harm reduction treatment given drinking levels were considerably lower than those before disulfiram initiation. A reduction in alcohol consumption can be reasonably expected to yield substantial benefits to the patient’s overall health and well-being [28, 29]. In the early 1960s, disulfiram was used in the framework of classical conditioning, being assumed to sensitize patients to alcohol [30] and promote cautious consumption [31], which has been deemed obsolete. In his autobiography, Per Olov Enquist describes how, despite the administration of disulfiram and its accompanying aversive reactions, he was able to resume regular alcohol consumption and even increase his intake over time [32]. Today it is widely accepted that the efficacy of disulfiram in reducing alcohol consumption cannot be attributed solely to its aversive effects. There is evidence [33, 34] suggesting that disulfiram might also reduce alcohol craving, potentially through the inhibition of dopamine β-hydroxylase, an enzyme implicated in the metabolism of dopamine and norepinephrine. This mechanism is also thought to contribute to disulfiram’s effects on cocaine dependence [35, 36]. The extent of craving reduction in the present cases remains unclear.

Our approach was possible due to comprehensive initial clinical workup, which are standard as a part of our treatment protocol, designed to identify and exclude any contraindications before initiating disulfiram. Key diagnostic measures included an exercise electrocardiogram (ECG) or comparable diagnostics to detect hemodynamically significant coronary artery disease. Moreover, routine laboratory evaluations were conducted, accompanied by an abdominal ultrasound for the exclusion of liver cirrhosis, and, when necessary, an esophagogastroduodenoscopy (EGD) to ascertain the absence of esophageal varices, which have the potential to pose significant, potentially life-threatening risks in instances of intense aversive reactions. Following the initial screening, any subsequent modifications in the patient’s condition necessitating a reassessment of disulfiram were subjected to rigorous monitoring via laboratory tests. These tests were designed to detect elevated liver enzymes as indications of liver damage. Additionally, ethylglucuronide (EtG) was analysed to draw inferences about the patient’s historical alcohol consumption. Furthermore, resting electrocardiograms (ECGs) were performed, if indicated, and clinical visits were conducted at designated intervals ranging between one and four weeks.

Despite these precautions, risks of disulfiram therapy with concurrent alcohol intake are hard to predict, even if aversive reactions are mild or not present. Elevated acetaldehyde levels, a metabolic product of the disulfiram-alcohol interaction, pose carcinogenic risks and are deemed to adversely affect multiple organs [37–40]. Given definitive threshold values and reliable biomonitoring methodologies are currently lacking [39], acetaldehyde concentrations during disulfiram therapy cannot be readily compared to those from prior episodes of heavy drinking. While elevated acetaldehyde levels act as a deterrent peripherally, they might paradoxically encourage alcohol consumption as they pass the blood-brain barrier and possibly cause psychotropic effects. In cases where reinforcing effects prevail over peripheral aversive effects, particularly in individuals with less active aldehyde dehydrogenase, disulfiram may even increase the risk of excessive alcohol consumption instead of protecting against alcoholism [41]. Beyond individual risks, disulfiram is typically administered in specialized treatment programs involving multiple patients who often interact and share their experiences, for instance, in treatment groups. Therefore, the chosen approach should not be disseminated to the patient population.

Both patients reported mild aversive disulfiram-alcohol reactions. Some individuals show no aversive disulfiram-alcohol reaction at all, which is often linked to insufficient dosing [42]. Disulfiram’s effectiveness as a prodrug can be compromised due to insufficient metabolism into its active form, diethyldithiocarbamic-acid-methyl ester (DDTC-Me), a process influenced by genetic variations in metabolic enzymes or other pharmacological factors [43]. However, adjustments to the dosage were not feasible, either due to patient refusal or because intermittent discontinuation made such changes ineffective.

We here report, to the best of our knowledge, the first known case reports in which disulfiram administration continued despite ongoing alcohol consumption by the patient.

The two case reports demonstrate that even in the context of continued alcohol use or positive laboratory evidence of alcohol consumption, maintaining disulfiram therapy warrants consideration when safer medications for reducing alcohol consumption, such as naltrexone or nalmefene, have failed in the past. Continuous prescribing may result in patients remaining in treatment for AUD and comorbid psychiatric disorders, thereby strengthening treatment adherence. However, this approach carries inherent risks associated with the acute disulfiram-alcohol reaction and chronic acetaldehyde exposure. By contrast, discontinuation of disulfiram therapy can cause treatment dropout, relapse to excessive alcohol consumption with detrimental health consequences and eventually also higher costs for the healthcare system. Thorough risk-benefit analyses conducted in collaboration with patients must ultimately reflect the necessity of individualized patient management strategies to support unique paths of recovery.

## Data Availability

The datasets used and/or analysed during the current study are available from the corresponding author on reasonable request.

## Abbreviations

ADR: Adverse drug reactions
ALAT: Alanine aminotransferase
APA: American Psychiatric Association
ASAT: Aspartate aminotransferase
AUD: Alcohol use disorder
DDTC-Me: Diethyldithiocarbamic-acid-methyl ester
ECG: Exercise electrocardiogram
EGD: Esophagogastroduodenoscopy
EMA: European Medicine Agency
EtG: Ethylglucuronide
FDA: Food and Drug Administration
NAP: Network of Alcohol-Aversive Pharmacotherapy
NICE: National Institute of Health and Care Excellence
PTSD: Post-traumatic stress disorder
RRD: Reduced-risk drinking
WHO: World Health Organization
